# Maternal PUFA ω-3 Supplementation Prevents Neonatal Lung Injuries Induced by Hyperoxia in Newborn Rats

**DOI:** 10.3390/ijms160922081

**Published:** 2015-09-14

**Authors:** Dyuti Sharma, Armande Subayi Nkembi, Estelle Aubry, Ali Houeijeh, Laura Butruille, Véronique Houfflin-Debarge, Rémi Besson, Philippe Deruelle, Laurent Storme

**Affiliations:** 1 EA 4489 Environnement Périnatal et Santé, Pôle Recherche Faculté de Médecine, Université Lille Nord de France, Lille 59045, France; E-Mails: dyuti.sharma@chru-lille.fr (D.S.); armande.subayi@chru-lille.fr (A.S.N.); estelle.aubry@chru-lille.fr (E.A.); ali.houeijeh@chru-lille.fr (A.H.); laura.butruille@hotmail.fr (L.B.); veronique.debarge@chru-lille.fr (V.H.-D.); remi.besson@chru-lille.fr (R.B.); philippe.deruelle@chru-lille.fr (P.D.); 2 Clinique de Chirurgie et Orthopédie de l’Enfant, Pôle Enfant, Hôpital Jeanne de Flandre, CHRU Lille, Lille 59037, France; 3 Clinique de Néonatologie, Pôle Femme, Mère et Nouveau-Né, Hôpital Jeanne de Flandre, CHRU Lille, Lille 59037, France; 4 Clinique de Gynécologie-Obstétrique, Pôle Femme, Mère et Nouveau-Né, Hôpital Jeanne de Flandre, CHRU Lille, Lille 59037, France

**Keywords:** PUFA ω-3, bronchopulmonary dysplasia, prematurity, diet

## Abstract

Bronchopulmonary dysplasia (BPD) is one of the most common complications of prematurity, occurring in 30% of very low birth weight infants. The benefits of dietary intake of polyunsaturated fatty acids ω-3 (PUFA ω-3) during pregnancy or the perinatal period have been reported. The aim of this study was to assess the effects of maternal PUFA ω-3 supplementation on lung injuries in newborn rats exposed to prolonged hyperoxia. Pregnant female Wistar rats (*n* = 14) were fed a control diet (*n* = 2), a PUFA ω-6 diet (*n* = 6), or a PUFA ω-3 diet (*n* = 6), starting with the 14th gestation day. At Day 1, female and newborn rats (10 per female) were exposed to hyperoxia (O_2_, *n* = 70) or to the ambient air (Air, *n* = 70). Six groups of newborns rats were obtained: PUFA ω-6/O_2_ (*n* = 30), PUFA ω-6/air (*n* = 30), PUFA ω-3/O_2_ (*n* = 30), PUFA ω-3/air (*n* = 30), control/O_2_ (*n* = 10), and control/air (*n* = 10). After 10 days, lungs were removed for analysis of alveolarization and pulmonary vascular development. Survival rate was 100%. Hyperoxia reduced alveolarization and increased pulmonary vascular wall thickness in both control (*n* = 20) and PUFA ω-6 groups (*n* = 60). Maternal PUFA ω-3 supplementation prevented the decrease in alveolarization caused by hyperoxia (*n* = 30) compared to PUFA ω-6/O_2_ (*n* = 30) or to the control/O_2_ (*n* = 10), but did not significantly increase the thickness of the lung vascular wall. Therefore, maternal PUFA ω-3 supplementation may protect newborn rats from lung injuries induced by hyperoxia. In clinical settings, maternal PUFA ω-3 supplementation during pregnancy and during lactation may prevent BPD development after premature birth.

## 1. Introduction

Bronchopulmonary dysplasia (BPD) is one of the most common complications of prematurity, occurring in 30% of very low birth weight infants [1]. This chronic lung disease results in high susceptibility of the preterm lung to injury during resuscitation, mechanical ventilation, and pro-inflammatory mediators that may disturb signaling pathways required for normal lung development. In two decades, neonatal medical care has improved, with antenatal steroids, surfactants, and less aggressive ventilation. The rate of severe lung injuries has decreased, giving way to a “new” form of BPD with alveolar hypoplasia and impaired vascular development. Current research has studied the persistent imbalance between pro-inflammatory and anti-inflammatory mechanisms in the development of “new BPD” in very low birth weight preterm infants [2,3,4]. Moreover, airway obstruction and respiratory symptoms persisted at school age in preterm children with a history of BPD compared to those without BPD [5].

The potential benefits of the dietary intake of polyunsaturated fatty acids ω-3 (PUFA ω-3) during pregnancy or the perinatal period have been reported [6,7], especially the effect of docosahexaenoic acid (DHA), accumulating in the brain and retina, on pre- and post-natal neural development [8]. Long-chain PUFA (LC-PUFA) ω-3 have important physiological properties during pregnancy, both for the pregnant woman and for the unborn child, and studies have shown that the placenta plays an important role in modulating its own fatty acids supply depending on fetal demands and is capable of preferentially transporting LC-PUFA to the fetal site [9]. Other studies have shown the possible benefits of feeding preterm infants with PUFA ω-3, including significant advantages in terms of growth and motor development [10]. More recently, a trend for decreased risk of BPD and reduction of risk of necrotizing enterocolitis was observed in preterm infants born at ≤32 weeks’ gestation exposed to long-chain PUFA ω-3 [11]. Some studies suggest the anti-inflammatory properties of long-chain PUFA ω-3 and explain the potential benefits of their use in dietary or in parental infusion to prevent inflammatory processes in clinical neonate care [12,13].

An animal model of BPD was developed on rodents because the birth of rodents occurs during the saccular stage of lung development. Hyperoxia exposure of newborn rats results in disruption of lung structure, affecting alveolarization and vascularization, a condition that strongly resembles “new BPD” in premature infants [1,14]. Lungs with “new BPD” present larger, simplified, and cystic alveoli, and irregular pulmonary vessels [15].

We hypothesized that maternal PUFA ω-3 supplementation during late pregnancy and the early postnatal period may prevent BPD injuries induced by hyperoxia in a model of newborn rats. The aim of this study was to assess the effects of maternal PUFA ω-3 supplementation, maternal PUFA ω-6 supplementation, and a maternal control diet on lung injuries in newborn rats exposed to hyperoxia, compared to those exposed to the ambient air.

## 2. Results

### 2.1. Effects of Hyperoxia on Survival and Growth of Newborns Rats and of Nursing Adult Rats

**Figure 1:**
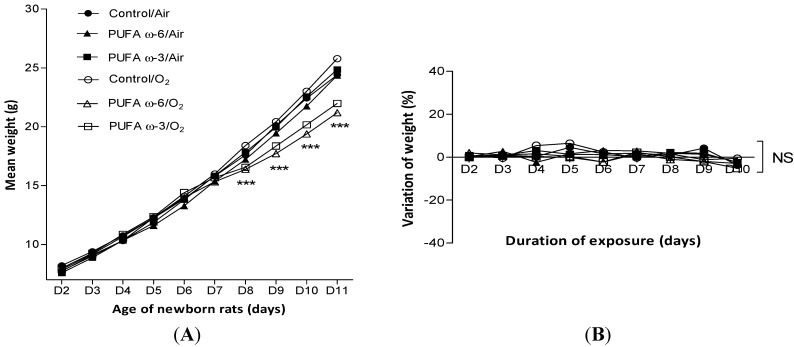
Evolution of weight of newborn rats (**A**) and daily variation of weight of nursing adult rats (**B**) depending on exposure to hyperoxia and type of supplementation. Value are expressed as mean ± SEM (*n* = 30 in PUFA ω-6/air, PUFA ω-3/air, PUFA ω-6/O_2_ and PUFA ω-3/O_2_, and *n* = 10 in control/air and control/O_2_). *** *p <* 0.001 comparing PUFA ω-6/O_2_ to control/air and to PUFA ω-6/air and comparing PUFA ω-3/O_2_ to control/air and to PUFA ω-3/air (Figure 1A). NS: not significant.

The survival rate was 100% in each group for newborn and adult rats. The mean weight of newborn rats exposed to hyperoxia was significantly lower than that of rats that were kept in the ambient air since their eighth (for PUFA ω-3/O_2_) or ninth (for PUFA ω-6/O_2_) day of life (Figure 1A). No difference was found between the daily variation in weight of nursing adult rats exposed to hyperoxia or not, or according to the type of PUFA supplementation (Figure 1B).

### 2.2. Effects of PUFA ω-3 on Lung Airway Development

**Figure 2:**
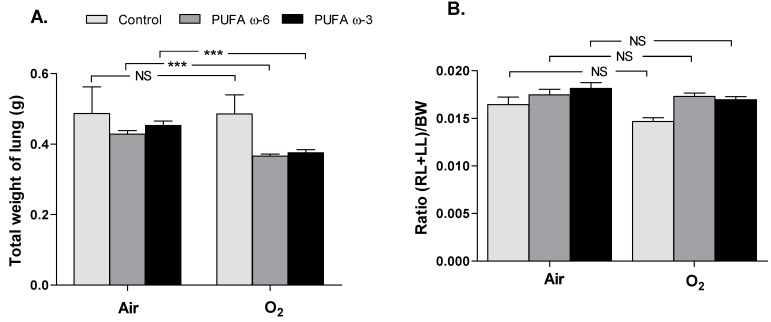
Lung morphometry, comparing the weight of both lungs (**A**) expressed as the ratio right and left lungs /body weights (RL + LL)/BW (**B**) in newborn rats exposed to hyperoxia or not, and according to the type of supplementation. Values are expressed as mean ± SEM (*n* = 30 in PUFA ω-6/air, in PUFA ω-3/air, in PUFA ω-6/O_2_ and in PUFA ω-3/O_2_, and *n* = 10 in control/air and in control/O_2_). *** *p <* 0.001; NS: not significant. RL: right lung; LL: left lung; BW: body weight.

The sum of the weights of the two lungs (in grams) of newborn rats exposed to hyperoxia (control/O_2_: 0.49 ± 0.06, PUFA ω-6/O_2_: 0.37 ± 0.01, PUFA ω-3/O_2_: 0.38 ± 0.01) was significantly lower (*p* < 0.001) (Figure 2A) than that of newborns kept in the ambient air (control/air: 0.49 ± 0.08, PUFA ω-6/air: 0.43 ± 0.01, PUFA ω-3/air: 0.45 ± 0.01), but the ratio of sum of both lungs weights/body weight ((RL + LL)/BW) was no different (Figure 2B).

**Figure 3:**
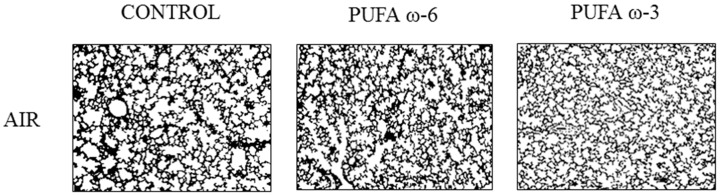

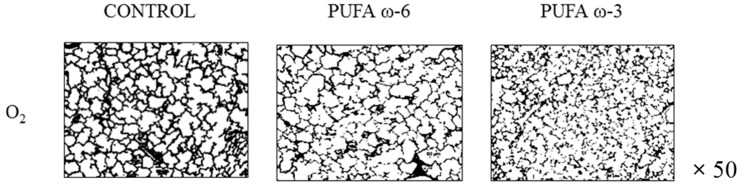
Lung sections showing impaired alveolization with decrease of septation and enlargement of distal airspaces in control/O_2_ and PUFA ω-6/O_2_; and increase of alveoli/fields and decrease of alveolar size in PUFA ω-3/O_2_. ×50: enlarge fifty times.

No lung injury was observed in newborn rats exposed to the ambient air. Lung histologic lesions observed in control group, exposed to 80%–85% FiO_2_, allowed us to validate the model of BPD. Impaired alveolization induced by hyperoxia was accompanied by a decrease in septation and an enlargement of distal airspaces in the control/O_2_ and PUFA ω-6/O_2_ groups (Figure 3). A more complex alveolar structure was noted in PUFA ω-3/O_2_ as the number of alveoli/fields increased and the alveolar size decreased (Figure 3).

**Figure 4:**
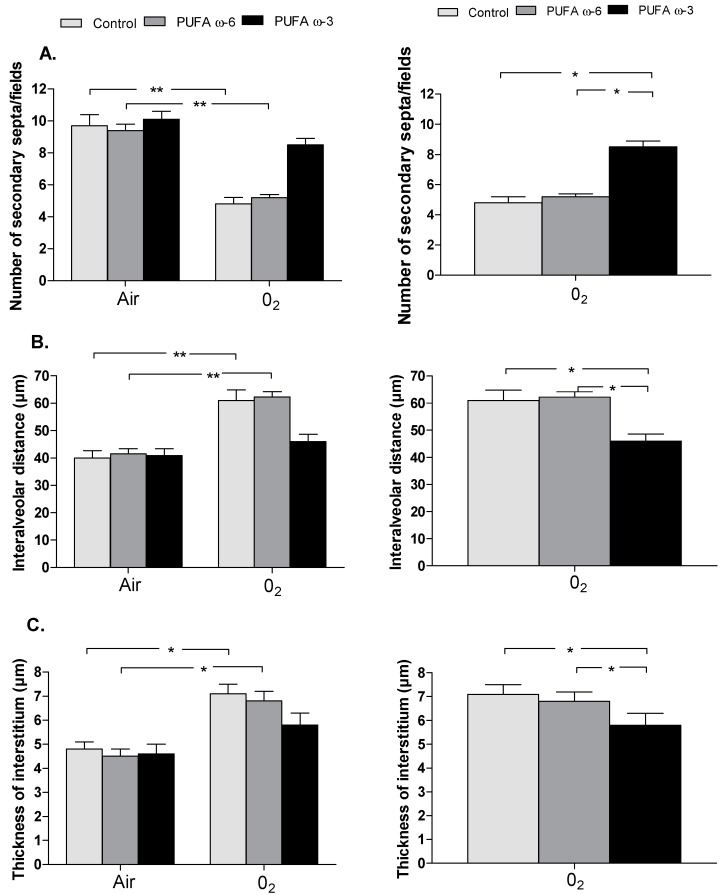
Lung morphometry, including the number of secondary septa/fields (**A**); the interalveolar distance (**B**); and the thickness of the interstitium (**C**). Value are expressed as mean ± SEM (*n* = 30 in PUFA ω-6/air, PUFA ω-3/air, PUFA ω-6/O_2_ and PUFA ω-3/O_2_ and *n* = 10 in control/air and control/O_2_). SEM: standard error of the mean; ** *p <* 0.01 and * *p <* 0.05.

Chronic exposure to hyperoxia was associated with a significant decrease in septation with halving of secondary alveolar septa (*p* < 0.01) in newborn rats of control/O_2_ (4.8 ± 0.4) and PUFA ω-6/O_2_ (5.2 ± 0.2) groups compared to control/air (9.7 ± 0.7) and PUFA ω-6/air (9.4 ± 0.4) (Figure 4A). We observed a significant increase in the mean interalveolar distance (in µm) (PUFA ω-6/air: 41.5 ± 1.9 *vs*. PUFA ω-6/O_2_: 62.2 ± 1.9 and control/air: 40 ± 2.6 *vs*. control/O_2_: 60.9 ± 3.9) of almost 50% (corresponding to enlargement of airspaces) (*p* < 0.01) (Figure 4B) and of the thickness of the interstitium (in µm) (PUFA ω-6/air: 4.5 ± 0.3 *vs*. PUFA ω-6/O_2_: 6.8 ± 0.4 and control/air: 4.8 ± 0.3 *vs*. control/O_2_: 7.1 ± 0.4) in newborn rats of control/O_2_ and PUFA ω-6/O_2_ groups compared to control/air and PUFA ω-6/air (*p* < 0.05) (Figure 4C).

PUFA ω-3 attenuates lung injuries induced by hyperoxia with no significant difference between the number of secondary alveolar septa/fields (PUFA ω-3/air: 10.1 ± 0.5 *vs*. PUFA ω-3/O_2_: 8.5 ± 0.4), the mean interalveolar distance (µm) (PUFA ω-3/air: 40.8 ± 2.6 *vs*. PUFA ω-3/O_2_: 46 ± 2.6), and the thickness of the interstitium (PUFA ω-3/air: 4.6 ± 0.4 *vs*. PUFA ω-3/O_2_: 5.8 ± 0.5) between the PUFA ω-3/O_2_ and PUFA ω-3/air groups (Figure 4A–C).

Maternal supplementation with PUFA ω-3 prevents lung injuries in pups exposed to hyperoxia compared to maternal supplementation with PUFA ω-6 and to the control group (Figure 4A–C), with a significant increase in the number of secondary septa/fields (PUFA ω-3/O_2_: 8.5 ± 0.4 *vs*. PUFA ω-6/O_2_: 5.2 ± 0.2, *p* < 0.05, PUFA ω-3/O_2_: 8.5 ± 0.4 *vs*. control/O_2_: 4.8 ± 0.4, *p* < 0.05). We observed a significant decrease in the mean interalveolar distance (PUFA ω-3/O_2_: 46 ± 2.6 *vs*. PUFA ω-6/O_2_: 62.2 ± 1.9, *p* < 0.05 and PUFA ω-3/O_2_: 46 ± 2.6 *vs*. control/O_2_: 60.9 ± 3.9, *p* < 0.05) and a decrease in the thickness of the interstitium (PUFA ω-3/O_2_: 5.8 ± 0.5 *vs*. PUFA ω-6/O_2_: 6.8 ± 0.4, *p* < 0.05 and PUFA ω-3/O_2_: 5.8 ± 0.5 *vs.* control/O_2_: 7.1 ± 0.4, *p* < 0.05).

### 2.3. Effects of PUFA ω-3 on Cardiovascular Development

**Figure 5:**
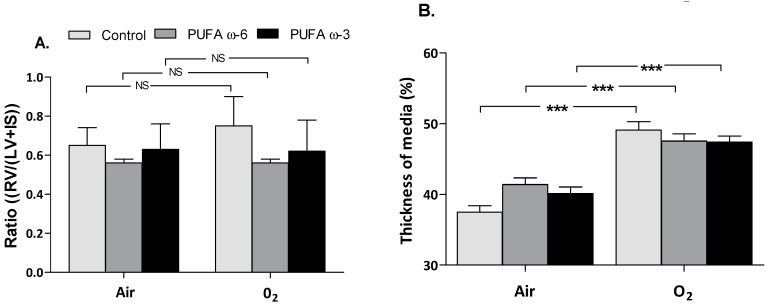
Heart and distal pulmonary arteries morphometry, comparing ratio (RV/(LV + IS)) in newborn rats exposed to hyperoxia or not (**A**) and the thickness of the media of distal pulmonary arteries (**B**) according to exposure and type of supplementation. Values are expressed as mean ± SEM (*n* = 30 in PUFA ω-6/air, PUFA ω-3/air, PUFA ω-6/O_2_ and PUFA ω-3/O_2_ and *n* = 10 in control/air and control/O_2_). RV: right ventricle; LV: left ventricle; IS: interventricular septum; *** *p* < 0.001; NS: not significant.

In our model, no right heart hypertrophy was identified. No significant difference was found regarding the ratio of weight of right ventricle/(weight of left ventricle + interventricular septum) (RV/(LV + IS)) between newborn rats exposed to hyperoxia and those kept in the ambient air (Figure 5A). Pulmonary vascular dysplasia with significantly increased thickness of media was shown in newborn rats exposed to hyperoxia, regardless of maternal diet supplementation (*p* < 0.001) (Figure 5B). No significant difference was found when comparing the mean thickness of media of PUFA ω-3/O_2_ to other groups of pups exposed to hyperoxia (*p* > 0.05).

## 3. Discussion

We showed that PUFA ω-3 supplementation in pregnant female Wistar rats prevents lung injuries in neonatal rat pups exposed to prolonged hyperoxia, in an *in vivo* model for experimental BPD. Our data demonstrate no difference between the number of secondary alveolar septa/fields, the mean interalveolar distance, and the thickness of the interstitium between the PUFA ω-3/O_2_ and PUFA ω-3/air groups. This study employed an experimental model of bronchopulmonary dysplasia described in many previous studies [14,15] and reproduced lung injuries in pup groups exposed to hyperoxia. In the same sequence, mean weight of pups exposed to hyperoxia was lower than those exposed to the ambient air, as described in studies using this BPD model [16,17].

Compared to maternal supplementation with PUFA ω-6, enriched PUFA ω-3 feeding to female pregnant rats exposed to hyperoxia could prevent development of BPD lung injuries in their broods. This result confirms the beneficial effects of infusion of PUFA ω-3 compared to infusion of PUFA ω-6 in lamb fetuses, as described in a previous work about the vascular effect on fetal pulmonary circulation [18]. Whereas vascular effects were well described in others experimental models [19,20], pulmonary tissue injuries but not vascular remodeling could be prevented by PUFA ω-3. In fact, all pup groups exposed to hyperoxia presented significant vascular injuries, compared to control groups (ambient air exposure) without the preventive effect of maternal supplementation of PUFA ω-3 or ω-6. Moreover, vascular injuries only concerned the thickness of the media of pulmonary arterioles and no right ventricular hypertrophy was described.

Common vascular injuries, as reported in similar studies about BPD experimental models [16,17,21], are arterial remodeling with increased thickness of the septum and arteriolar wall, and cardiac consequences such as right ventricular hypertrophy and pulmonary hypertension. However, the occurrence of this type of lesion seemed to correlate both with the degree (FiO_2_ %) and the duration of hyperoxia exposure and of “recovering” time, and, in our study, early lung morphometric analysis at Day 11 of exposure could explain why only arterial remodeling was found. The duration of exposure to hyperoxia was the same as in these other studies, but in the other studies pups were given a recovery period of a few days in the ambient air after exposure, during which some lung and vascular injuries were fixed. Moreover, we can hypothesize that maternal supplementation with PUFA ω-3 was sufficient to prevent the development of lung injuries but not long enough in duration to have vascular effects on arterial remodeling.

Many studies [8,11] have demonstrated the potential benefits of a diet enriched with PUFA ω-3 in the neonatal period. These benefits, especially for the neurodevelopmental growth and acuity of newborns, were studied in pregnant women [6]. The present study found a preventative effect of maternal dietary supplementation with PUFA ω-3 on potential BPD injuries in an experimental model of lung injuries by hyperoxia exposure, but has not allowed us to pinpoint the length of time necessary for preventive action against BPD injuries. We decided to start PUFA supplementation before the end of gestation and to continue during lactation because the benefits of PUFA are well described in the perinatal period, especially in the prevention of prematurity and thus also in the prevention of BPD. This is why PUFA ω-3 supplementation is actually preconized during pregnancy and lactation.

Many studies have described the potential pathologic pathways of “new BPD” injuries and lung injuries after hyperoxia exposure. The production and increase of chemokines and pro-inflammatory cytokines TNFα, IL-1β, IL-6, and IL-8 could be involved in the development of BPD. Another pathway described is p38, a member of the MAKP family also related to the JNK pathway (c-Jun NH_2_-Terminal Kinase), which involves stress-activated protein kinases. Activation of the p38 and JNK pathway leads to apoptosis of lung cells and lung macrophages [22]. A review of the literature about inflammatory biomarkers in premature neonates [3] found increased levels of pro-inflammatory cytokines (IL-8, IL-1, TNF α) in cases of BPD. The benefits of PUFA ω-3 on the balance of pro- and anti-inflammatory cytokines has been well described. More recently, a study of inflammation and vascular remodeling in pulmonary hypertension induced by monocrotaline in adult rats [23] found a decrease of NF-κB and p38 MAKP with less inflammation and vascular remodeling in the group treated with MAG-DPA (docosapentaenoic acid monoacylglycerid), a metabolite of EPA (eicosapentaenoic acid) and DHA (docosahexaenoic acid), compared to the control group. Furthermore, a study on the dosage of inflammation biomarkers after heart surgery in the neonatal period [12] found a significant decrease of pro-inflammatory markers (TNFα, IL-1β and IL-6) in the plasma of newborns treated before and after surgery with lipid emulsion containing EPA and DHA. Another hypothesis of the mechanism of action would be mediated by resolving D1 and lipoxin A4, terminal derived metabolites of DHA and arachidonic acid, respectively. A recent study [24] demonstrated that preventive treatment with these metabolites of DHA and arachidonic acid reduces the number of BPD lung injuries in mice pups exposed to hyperoxia. These data suggest that two potential mechanisms of action of PUFA ω-3 supplementation are regulation and decrease of pro-inflammatory biomarkers involved in the inflammation pathways of “new BPD” injuries.

Our study has some limitations. The present study is a pilot study about the effects of maternal supplementation on the development of BPD in offspring. At the time of this study, no data were available to inform our sample size calculations. The choice of 30 pups per treatment group was made arbitrarily. The control group was smaller than the other groups, which represents a limitation of the present study. No impact of gender has been described for bronchopulmonary dysplasia in the literature. Because there is no sex ratio in BPD, the gender of the pups was not collected and no sex grouping was done. PUFA dietary intake was monitored daily in pregnant rats by feeding them with 0.2 mL of Omacor^®^ or sunflower oil with 0.8 mL of water, from the 14th day of gestation to the 10th day after delivery. Pups were fed by breastfeeding. The PUFA intake of pups has not been assessed and thus represents another limitation of the study.

Because of the small sample of control animals (*n* = 3 litters for the PUFA ω-3/O_2_, PUFA ω-6/O_2_, PUFA ω-3/air, and PUFA ω-6/air groups and *n* = 1 litter for the control/O_2_ and control/air groups), we have not be able to perform multiparametric statistic tests to study the variable “litter” and we analyzed data with one-way ANOVA and *post hoc* Bonferroni tests.

The present study allows us to suggest that maternal feeding with PUFA ω-3 can prevent BPD injuries from developing in newborns with a high risk of prematurity. PUFA ω-3 supplementation has recently been studied in human clinics in the situation of severe preterm neonates with respiratory distress syndrome [25]. It could be interesting to investigate and study the impact of maternal feeding with PUFA ω-3 in the situation of congenital malformation of the diaphragm such as congenital diaphragmatic hernia, which is often associated with a high risk of lung hypoplasia and BPD development.

## 4. Experimental Section

### 4.1. Animals

All animal procedures and protocols used in this study were performed in the Department of Experimental Research at our Hospital Center (Animal experimentation agreement n 59286) and were approved by the French “Ministère de l’Agriculture, de la Pêche et de l’Alimentation” before the studies were conducted.

Fourteen pregnant Wistar rats from Janvier Laboratory Animals (Janvier SAS, St Berthevin, France) were housed in our laboratory for seven days of gestation. Pregnant rats were fed *ad libitum* with the standard diet SAFE A04 (59.9% of carbohydrates, 16.1% of protein, 11.9% of water, 3.1% of lipids, including 54% of ω-6 polyunsaturated fatty acid, 21% saturated fatty acids, and 17% monounsaturated fatty acids) and exposed to a day/night cycle of 12 h alternatively.

### 4.2. Experimental Design

After one week of acclimatization, pregnant rats were fed daily on 0.2 mL of PUFA ω-3 (Omacor^®^ (*n* = 6): 158 mg (462 mg·kg^−1^·d^−1^) of LC PUFA per day, corresponding to 225 mg·kg^−1^·d^−1^ of EPA, 187 mg·kg^−1^·d^−1^ of DHA, and 50 mg·kg^−1^·d^−1^ of α linolenic acid with ratio EPA/DHA of 1.20), with 0.2 mL of PUFA ω-6 (sunflower oil (*n* = 6): 120 mg of LC PUFA per day corresponding to 350 mg·kg^−1^·d^−1^ of linoleic acid) with 0.8 mL of water (to flush feeding tube), or with 1 mL of water (control group, *n* = 2) since 14th day of gestation to day 10 after delivery. Birth occurred spontaneously on the 21st day of gestation and pups were pooled to balance groups with 10 pups and nursing adult rats. At 36 h of life (adaptation to extrauterine life [26]), newborn and adult rats were exposed continuously (23.5 h/24 h) using a sealing and plastified oxygen tent (Vygon^®^, Ecouen, France) to hyperoxia (O_2_ groups, oxygen between 80% and 85%), with partial pressure of carbon dioxide less than 1 mmHg [27] or not (air ambient group, oxygen 21%) over 10 days. The flow of gas was 5 L/m. The concentration of oxygen was continuously measured by an oxygen monitor (Airox- BioMS^®^, Pau, France) and the partial pressure of CO_2_ was monitored with a spectrophotometric analyzer (Novametrix/Respironics/Philips^®^, Solna, Sweden). The temperature of the chamber was continuously monitored. For only 30 min per day, the chambers were opened for the feeding and weighing of animals. Pups were fed by breastfeeding, whether they were exposed to hyperoxia or not.

At this level of study, six groups of newborn rats were obtained: PUFA ω-6/O_2_ (*n* = 30), PUFA ω-6/air (*n* = 30), PUFA ω-3/O_2_ (*n* = 30), PUFA ω-3/air (*n* = 30), control/O_2_ (*n* = 10), and control/air (*n* = 10). The survival rate and weight of newborn rats were recorded daily during the study.

### 4.3. Tissue Preparation and Histomorphometric Analysis

All animals were sacrificed at day 11 after birth. Weights of total body, lungs, and heart were taken. Lungs were prepared just after sternotomy by tracheal cannulation with a 24 G tube for intratracheal instillation of paraformaldehyde (4% PAF) at a constant pressure of 30 cm H_2_O for 30 min, after which the trachea was ligated. The heart-lungs unit was removed and placed into 4% PAF for 12 h. Finally, the fixed lungs were embedded in paraffin. A cross section of 5 µm thickness was taken from the three right lung lobes.

Histological slides were stained with hematoxylin and eosin to evaluate the alveolar structure and vascular remodeling. Histological slides were prepared with a microscope (Axiophot 2-Zeiss, Jena, Germany) combined with a numeric camera (Axiocam IC-Zeiss, Jena, Germany) and processed using Axiovision software (Zeiss, Jena, Germany) with 50× enlargement, saved into TIFF high-resolution format and then used with ImageJ software (image processing software created by Mr. Wayne Rasband, National Institute of Mental Health, Bethesda, MD, USA, available at http://rsb.info.nih.gov/ij).

### 4.4. Main Parameters of Study

The general parameters were:

Survey rate and evolution of weight gain of newborn rats; survey and daily variation of weight of nursing adult rats; the histological and morphometric parameters were: weight of lungs to compute the ratio: ratio = (weight of right + left lungs)/body weight ((RL + LL)/BW) to assess pulmonary growth; lung alveolization evaluated by measurement of mean interalveolar distance [26], by number of secondary septas/fields, and by measurement of thickness of interstitium; weight of right ventricle and history of right heart hypertrophy by calculating: ratio = weight of right ventricle/(weight of left ventricle + interventricular septum) (RV/(LV + IS)) [28]; external diameter of vessels and thickness of wall of distal pulmonary arterials (size between 10 and 50 µm) to search for hypertrophy of media calculated by: thickness of media = (2 × wall thickness × 100)/external diameter) [28].

### 4.5. Statistical Analysis

Values were expressed as mean ± SEMs. The comparisons between groups were analyzed with an analysis of variance test followed by one-way or repeated ANOVA, and a Bonferroni test was performed for multiple comparison using software Graphpad Prism version 5^®^ (Graphpad Software, Inc., San Diego, CA, USA). Statistical tests were used to compare pups’ growth and morphometric measures in each maternal supplementation group exposed to hyperoxia *versus* the ambient air. Differences with a *p* value of less than 0.05 were considered statistically significant.

## 5. Conclusions

In conclusion, PUFA ω-3 prevented the development of chronic lung disease in premature newborns in an experimental animal model. These results are pre-clinical prerequisites and should be confirmed with a prospective study in a human clinic on the effect of maternal dietary supplementation with PUFA ω-3 on BPD occurrence in newborns.
